# Correction: Perspectives of multidisciplinary healthcare providers in elderly daycare centres: Challenges, opportunities and impacts on geriatric care in Chiangrai Municipality, Thailand

**DOI:** 10.1371/journal.pone.0344326

**Published:** 2026-03-04

**Authors:** Paralee Opasanant, Panitsara Leekuan

The first author’s name is spelled incorrectly. The correct name is: Paralee Opasanant. The correct citation is: Opasanant P, Leekuan P (2025) Perspectives of multidisciplinary healthcare providers in elderly daycare centres: Challenges, opportunities and impacts on geriatric care in Chiangrai Municipality, Thailand. PLoS One 20(9): e0331453. https://doi.org/10.1371/journal.pone.0331453
