# Supplementary figures and images for: Perspectives of multidisciplinary healthcare providers in elderly daycare centres: Challenges, opportunities and impacts on geriatric care in Chiangrai Municipality, Thailand
Source: PLoS One. 2025 Sep 19;20(9):e0331453. doi: 10.1371/journal.pone.0331453 (PMC12449021; doi:10.1371/journal.pone.0331453)

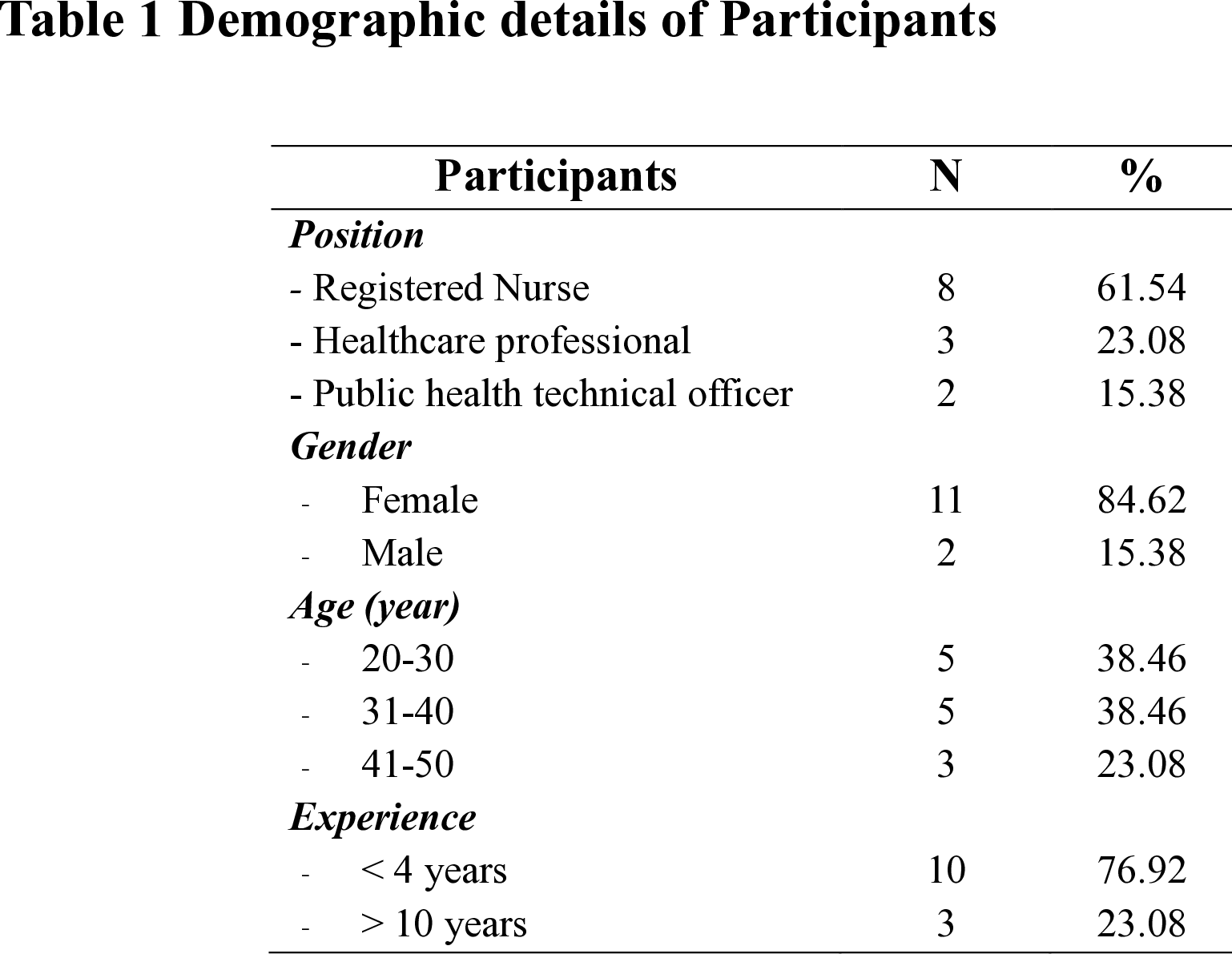

Supplement: S1 Table — (TIF) [file pone.0331453.s002.tif]
