# Supplementary material for: Perspectives of multidisciplinary healthcare providers in elderly daycare centres: Challenges, opportunities and impacts on geriatric care in Chiangrai Municipality, Thailand
Source: PLoS One. 2025 Sep 19;20(9):e0331453. doi: 10.1371/journal.pone.0331453 (PMC12449021; doi:10.1371/journal.pone.0331453)
Supplement: Attachment — Submitted filename: Response to Reviewers II.pdf [file pone.0331453.s005.pdf]

Outline for revising Manuscript

Perspectives of Multidisciplinary Healthcare Providers in Elderly Daycare Centres: Challenges, Opportunities and Impacts on Geriatric Care in Chiangrai Municipality, Thailand

Original version		Revision	Page and Line
**Journal Requirements:**	1. Please review your reference list to ensure that it is complete and correct.	• All references are rechecked in order to complete and correct.<br>• Reference no. 3 is revised.<br>• Reference no. 13 is revised.	Page 31-32
**Method: Data Analysis**	2. Clarify Method Blending: Distinguish IPA from Thematic Analysis or Justify Hybrid. Please confirm in the method section.	• This study employed Interpretative Phenomenological Analysis (IPA) as its qualitative methodology in order to explore the lived experiences and meaning-making processes of participant group in depth. Unlike more general approaches to thematic analysis which identify overarching themes across a dataset.<br>• IPA's focus on hermeneutic interpretation provided the philosophical underpinning for this in-depth engagement with each participant's narrative	Page 8,9<br>Line 243-275
**Introduction & Discussion**	3. Clarified Theoretical Positioning (Suggested Section: Introduction & Discussion) such as integrated Activity Theory, Person-Centered Care PCC, and Organizational Support Theory OST	Introduction:<br>• Added Theoretical theory: Integrated Activity Theory, Person-Centered Care (PCC) and Organizational Support Theory (OST) in Introduction section	Page 4-5<br>Line 110-147

Original version		Revision	Page and Line
		Discussion:<br>• Added Theoretical theory: Integrated Activity Theory, Person-Centered Care (PCC) and Organizational Support Theory (OST) in Discussion section	Page 21-22<br>Line 620-647<br><br>Page 25-26<br>Line 742-764<br><br>Page 26-27<br>Line 787-796,<br>Line 803-813
	4. Applied to interpret how institutional structures shape staff motivation and collaborative effectiveness.	• Added the organizational structure facilitated robust interprofessional collaboration, which, in turn, boosted motivation.	Page 16<br>Line 457-470
**Data Analysis**	5. Synthesized participant narratives into conceptual insights in analysis such as Healthcare providers interpreted government mandates not as abstract directives but as dynamic frameworks that guide clinical practice.<br><br>Weekly planning and debriefing meetings created a cyclical feedback loop, enhancing implementation fidelity.<br><br>Providers saw themselves as "conduits of policy," translating structural goals into person-centered routines. This aligns with Organizational Support Theory, as	• Added information about Healthcare providers viewed government commands as adaptable frameworks that informed their daily clinical practice, not as inflexible, theoretical regulations.<br><br>• Added Structured Weekly meetings to support adaptive care delivery<br><br>• Added the core tenets of OST, which support organization values and cares	Page 12<br>Line 329-336<br><br>Page 13<br>Line 388-394<br><br>Page 16<br>Line 457-470

Original version		Revision	Page and Line
	structured reflection improved morale and perceived relevance of their work		
	6. Enhance Visual Representation: Include Conceptual Framework or Model	• Included Conceptual Framework for Elderly Daycare Centre	Page 29<br>Figure 1
