# Supplementary material for: Perspectives of multidisciplinary healthcare providers in elderly daycare centres: Challenges, opportunities and impacts on geriatric care in Chiangrai Municipality, Thailand
Source: PLoS One. 2025 Sep 19;20(9):e0331453. doi: 10.1371/journal.pone.0331453 (PMC12449021; doi:10.1371/journal.pone.0331453)
Supplement: Attachment — Submitted filename: PONE-D-25-14917_reviewer comments.docx [file pone.0331453.s003.docx]

Thank you for the opportunity to review the manuscript “Perspectives of Multidisciplinary Healthcare Providers in Elderly Daycare Centres: Challenges, Opportunities and Impacts on Geriatric Care in Chiangrai Municipality, Thailand”. The manuscript explores the lived experiences of healthcare providers delivering care within community-based elderly daycare settings. The authors aim to identify key challenges healthcare professionals face, successful practices utilized, and potential opportunities for enhancing geriatric care services through qualitative inquiry. The study used a phenomenological approach to gather data from twelve multidisciplinary providers, including registered nurses, physiotherapists, nutritionists, and public health technical officers, through semi-structured interviews. Data analysis involved thematic and interpretative phenomenological analysis (IPA), revealing four central themes: opportunities in policy implementation, challenging experiences, enhancement of organizational effectiveness, and reflections on program outcomes and impacts. The manuscript highlights essential insights into how multidisciplinary teams perceive and experience geriatric care services, underscoring the significance of integrated, community-oriented care models for older adults. However, numerous critical areas must be addressed to strengthen the manuscript's academic rigor, theoretical coherence, and methodological clarity, as below:

1. The theoretical perspectives are vaguely described and analyzed:

- The manuscript does not explicitly articulate a guiding theoretical approach, essential in phenomenological research. As a qualitative methodology, phenomenology seeks to deeply explore individuals' lived experiences to uncover the meanings and essences of phenomena as perceived by the participants. However, the manuscript does not specify whether the phenomenological approach employed aligns with Husserl's descriptive phenomenology, focusing on bracketing researchers' assumptions to reveal the pure essence of experiences, or Heidegger's interpretative phenomenology (hermeneutic phenomenology), which emphasizes the researchers' interpretative engagement with the data and recognizes their preconceived notions as integral to interpretation. The authors are, therefore, encouraged to articulate the theoretical perspectives underpinning their phenomenological research explicitly and then clarify how phenomenology directly shapes data collection procedures, the process of conceptualization, and subsequent interpretation of findings.
- In data gathering, specifying the chosen phenomenological approach would better guide and justify the construction of interview questions, the conduct of interviews, and the researchers’ role within these interactions. The authors might benefit from explicitly describing how they managed their assumptions during interviews (through bracketing or interpretive engagement) to enhance transparency and methodological rigor.
- Regarding conceptualization, it is recommended that the authors clearly describe the process used to identify and categorize meaningful statements and themes. A detailed explanation of whether descriptive or interpretative phenomenological principles guided this would strengthen the credibility and clarity of theme development.
- When interpreting findings, it is advisable to explicitly acknowledge the hermeneutic process, particularly if adopting Heidegger’s interpretative phenomenology. Describing the iterative process of interpretation—the "hermeneutic circle"—would substantially enhance the depth and transparency of the findings. Clearly defining the theoretical approach will significantly improve the manuscript’s theoretical coherence, methodological rigor, and interpretive clarity.

2. The semi-structured interviews are suitable for qualitative phenomenological research, yet the development and validation of the interview guide lack sufficient detail. The authors should clearly describe the process of constructing, piloting, and refining the interview guide, thereby enhancing methodological transparency and ensuring content validity
3. The data analysis approach combining thematic and interpretative phenomenological analysis (IPA) is appropriate; however, the manuscript lacks sufficient procedural details that assure methodological rigor. Detailed accounts of specific practices such as member-checking, peer debriefing, reflexivity strategies, and inter-rater reliability would considerably improve analytical robustness. A clear reflexivity statement addressing the researchers' positionality and potential biases would ensure transparency and enhance credibility.
4. According to the findings, while the identified themes are relevant and informative, they could benefit from deeper theoretical interpretation. The manuscript presents themes descriptively but misses the opportunity to explicitly connect with existing aging theories, stress-coping mechanisms, or organizational behavior theories. Such theoretical integration would enrich the interpretation and increase the manuscript's scholarly value.
5. The implications for practice and policy are implicitly stated but require more precise articulation. The manuscript would benefit significantly from explicitly outlining actionable policy recommendations and practical interventions derived from the findings. Additionally, discussing the potential scalability of identified successful practices across broader contexts would add practical relevance.
6. A comprehensive discussion of limitations, particularly regarding the transferability of findings, would strengthen the manuscript. Explicitly addressing contextual limitations (cultural, regional, and organizational) and suggesting pathways for future research would enhance methodological rigor and academic value.
7. The Introduction section of the manuscript provides comprehensive context and thorough background information on elderly care and daycare centers. However, it is overly detailed and somewhat repetitive, diluting the reader's focus on the study's objectives and significance. I recommend condensing this section by succinctly synthesizing relevant demographic data and policy contexts. Clarify and streamline the narrative by integrating similar ideas, particularly regarding the aging population's implications, healthcare system pressures, and policy frameworks. This conciseness will enhance readability and emphasize the core rationale and objectives of the study.
8. The authors have provided detailed quotes from participants that significantly enrich the manuscript by illustrating the lived experiences of multidisciplinary healthcare providers in elderly daycare centres. However, the presentation of quotes could be optimized for clarity and effectiveness. Specifically, it is recommended that the authors critically evaluate and selectively present quotes to ensure they succinctly reflect key themes and avoid redundancy. The manuscript would benefit from more strategically integrated quotes, emphasizing those that most strongly illustrate the core findings or unique insights relevant to the research objectives. Overly lengthy quotes could be condensed to maintain reader engagement and highlight only the most pertinent data points. The authors will enhance their qualitative findings' overall readability and impact by carefully curating and refining the quoted content.

In conclusion, the manuscript offers meaningful contributions to understanding multidisciplinary geriatric care experiences but requires improvements in theoretical clarity, methodological rigor, and interpretive depth. Addressing these points with MAJOR REVISION would significantly enhance its suitability for publication in PLOS ONE.
