# Supplementary material for: Perspectives of multidisciplinary healthcare providers in elderly daycare centres: Challenges, opportunities and impacts on geriatric care in Chiangrai Municipality, Thailand
Source: PLoS One. 2025 Sep 19;20(9):e0331453. doi: 10.1371/journal.pone.0331453 (PMC12449021; doi:10.1371/journal.pone.0331453)
Supplement: Attachment — Submitted filename: Response to Reviewers.docx [file pone.0331453.s004.docx]

**Outline for revising Manuscript**

**Perspectives of Multidisciplinary Healthcare Providers in Elderly Daycare Centres: Challenges, Opportunities and Impacts on Geriatric Care in Chiangrai Municipality, Thailand**

| **Original version** | | **Revision** | | **Page and Line** |
| --- | --- | --- | --- | --- |
| **The comments** | | - Articulated phenomenological study Heidegger’s phenomenology and interpretive phenomenology, emphasize understanding the providers’ interpretations of their experiences - **Data collection methods** were designed to facilitate deep, rich narratives through semi-structured interviews, open-ended narrative accounts and reflective journaling. that captures the essence of lived experiences. - **Data analysis** reflected on how the researchers’ interpretations were shaped by their engagement with the data, ensuring that the essence of participants’ experiences remains central. | | Page 2  Line 33-37  Page 6  Line 167-175  Page 7  Line 205- 219 |
| **The theoretical perspectives** | 1. Encouraged to articulate the theoretical perspectives underpinning their phenomenological research explicitly 2. Clarify how phenomenology directly shapes data collection procedures, the process of conceptualization, and subsequent interpretation of findings |  |  |  |
|  | 3. The authors might benefit from explicitly describing how they managed their assumptions during interviews (through bracketing or interpretive engagement) to enhance transparency and methodological rigor. |  | - Added detailed of management geriatric care model before conducted the study - The interview summaries were shared with participants to ensure the interpretations matched their intended meanings. - All interviews were compared and reviewed interpretations with another researcher. | Page 6  Line 185-195  Page 8  Line 249-251  Page 8  Line 240-242 |
|  | 4. A detailed explanation of whether descriptive or interpretative phenomenological principles guided this would strengthen the credibility and clarity of theme development.   |  | - Revised Interpretative Phenomenological Analysis in order to enhance credibility through transparency. - The rigour of the study was written to illustrate transparency and to establish the trustworthiness of the findings through strategies such as triangulation, reflexivity, and detailed documentation of the research process. | Page 7-8  Line 205-242  Page 9-10  Line 243-278 |
|  | 5. Describing the iterative process of interpretation—the "hermeneutic circle"—would substantially enhance the depth and transparency of the findings. 6. Clearly defining the theoretical approach will significantly improve the manuscript’s theoretical coherence, methodological rigor, and interpretive clarity.   |  | - This study employed interpretative phenomenological analysis (IPA), with particular emphasis on the hermeneutic circle as a methodological cornerstone. - Added using the hermeneutic circle central to Interpretative Phenomenological Analysis (IPA), | Page 8  Line 212-242 |
| **Introduction** | 7. it is overly detailed and somewhat repetitive, diluting the reader's focus on the study's objectives and significance. I recommend condensing this section by succinctly synthesizing relevant demographic data and policy contexts. |  | - Condensed the section | Page 3-5  Line 58-126 |
|  | 8. Clarify and streamline the narrative by integrating similar ideas, particularly regarding the aging population's implications, healthcare system pressures, and policy frameworks.   This conciseness will enhance readability and emphasize the core rationale and objectives of the study |  | - Added in Conclusion | Page 24  Line 714-730 |
| **Methods** | 9. the development and validation of the interview guide lack sufficient detail. The authors should clearly describe the process of constructing, piloting, and refining the interview guide, thereby enhancing methodological transparency and ensuring content validity |  | - We have developed this section and included more information about the study design, recruitment and analysis in the location show. | Page 6  Line 167-174 |
|  | 10. Since your study includes only one physiotherapist and one nutritionist, could coding by profession reveal the identity of the participants? You should consider this carefully. |  | - Broader professional categories: Refer to participants as "healthcare professionals" rather than specific roles | Page 10  Line 285  Table 1 |
| **Study design** | 11. Require improvements methodological rigor, and interpretive depth |  | - Methodological rigor:   Included: (1) methodological memos detailing procedural decisions; (2) analytical memos tracking the evolution of codes and themes; (3) reflexive journals examining researcher positionality; and (4) team deliberation logs capturing interpretive dialogues among researchers. and the methodological integrity of our approach.   - Analysis:   Added interpretive approach moved beyond thematic categorization to achieve analytical depth through systematic application of multiple interpretive lenses.   - Added, reflexive journaling documented our evolving relationship with the data, tracking moments of insight, confusion, or resonance that shaped interpretive processes. | Page 5  Line 129-141  Page 7  Line 181-204  Page 7  Line 206-242  Page 6-7  Line 179-203 |
|  | 12. The description of activities within the care model at the study centers is insufficiently detailed.   Could the author provide a rationale for the purposive sampling method employed, specifically addressing the exclusion of physicians? |  | - Added a rational for Purposive sampling in Methods setting - Added the exclusion of physicians from our sampling frame. This is because physicians primarily function as external consultants to elderly daycare centers rather than integrated team members, with physician visits occurring episodically rather than as part of daily operations. Their experiences fundamentally differed from those of the core multidisciplinary team who navigate daily interprofessional interactions within these | Page 5  Line 135-141  Page5  Line 148-149 |
|  | 13. the manuscript lacks sufficient procedural details that assure methodological rigor. Detailed accounts of specific practices such as member-checking, peer debriefing, reflexivity strategies, and inter-rater reliability would considerably improve analytical robustness. A clear reflexivity statement addressing the researchers' positionality and potential biases would ensure transparency and enhance credibility |  | - To addressed about Reflexivity | Page 7  Line 181-204 |
|  | 14. The manuscript presents themes descriptively but misses the opportunity to explicitly connect with existing aging theories, stress-coping mechanisms, or organizational behavior theories.   Such theoretical integration would enrich the interpretation and increase the manuscript's scholarly value. |  | - added in Implicatons for practice - the opportunity to explicitly connect with existing aging theories, stress-coping mechanisms, or organizational behavior theories | Page 25  Line 731-738 |
|  | 15. The presentation of quotes could be optimized for clarity and effectiveness. Specifically, it is recommended that the authors critically evaluate and selectively present quotes to ensure they succinctly reflect key themes and avoid redundancy. The manuscript would benefit from more strategically integrated quotes, emphasizing those that most strongly illustrate the core findings or unique insights relevant to the research objectives |  | - Quotes were illustrated and integrated to ensure they succinctly reflect key themes relevant to the research objectives | Page 11-18  Line 300-536 |
|  | 16. Overly lengthy quotes could be condensed to maintain reader engagement and highlight only the most pertinent data points. The authors will enhance their qualitative findings' overall readability and impact by carefully curating and refining the quoted content. |  | - Condensed quotes | Page 11-18  Line 300-536 |
|  | 17. Title "Pharmacist," in line 378 but it is not described in Demographic details of participants. Is there an error here |  | - Added in the details of participants | Table 1 |
| **Discussion** | 18. Explicitly addressing contextual limitations (cultural, regional, and organizational) and suggesting pathways for future research would enhance methodological rigor and academic value. |  | - To address contextual limitations (cultural, regional, and organizational) - To address Further research | Page 25  Line 739-750  Page 25  Line 734-738 |
| **The implications for practice and policy** | 19. The manuscript would benefit significantly from explicitly outlining actionable policy recommendations and practical interventions derived from the findings. Additionally, discussing the potential scalability of identified successful practices across broader contexts would add practical relevance. |  | - To outline actionable policy recommendations and practical interventions derived from the findings - To discuss the potential scalability of identified successful practices across broader contexts would add practical relevance | Page 25  Line 731-738 |
